# Cross-sectional examination of musculoskeletal conditions and multimorbidity: influence of different thresholds and definitions on prevalence and association estimates

**DOI:** 10.1186/s13104-017-2376-4

**Published:** 2017-01-18

**Authors:** Dianne B. Lowe, Michael J. Taylor, Sophie J. Hill

**Affiliations:** 1 Centre for Health Communication and Participation, School of Psychology and Public Health, College of Science, Health and Engineering, La Trobe University, Melbourne, Australia; 2 School of Allied Health, Australian Catholic University, Fitzroy, Australia; 3 Cochrane Consumers and Communication Review Group, School of Psychology and Public Health, College of Science, Health and Engineering, La Trobe University, Melbourne, Australia

**Keywords:** Multimorbidity, Comorbidity, Musculoskeletal conditions, Prevalence

## Abstract

**Background:**

Multimorbidity and musculoskeletal conditions create substantial burden for people and health systems. Quantifying the extent of co-occurring conditions is hampered by conceptual heterogeneity, imprecision and/or indecision about how multimorbidity is defined. The purpose of this study is to examine the influence of different ways of operationalising multimorbidity on multimorbidity prevalence rates with a focus on working-age adults with musculoskeletal conditions. Weighted population prevalence rates of multimorbidity among working-age Australians were estimated using data from the National Health Survey. Two nominal thresholds (2+ or 3+ co-occurring conditions) and three operational definitions of multimorbidity (survey-, policy- and research-based) were examined. Using logistic regression, we estimated the association between the prevalence of multimorbidity among persons with musculoskeletal conditions compared to persons with non-musculoskeletal conditions for each definition and threshold combination.

**Results:**

As few as 7.9% of working-age Australians have 2+ conditions using the research-based definition (95% CI 7.4–8.5%), compared to estimates of 15.3% (95% CI 14.3–16.2%) and 61.5% (95% CI 60.3–62.7%). with the policy- and survey-based definitions, respectively. Depending on definition, with the 3+ threshold multimorbidity prevalence ranged from 2.1% (research) to 41.9% (survey). Among the sub-sample with musculoskeletal conditions, multimorbidity with the 2+ threshold ranged from 20.2 to 92.2%; and with 3+ threshold from 5.9 to 75.4%, again lowest with the research-definition and highest with the survey-definition. When compared to any other condition (i.e. non-musculoskeletal conditions), all musculoskeletal conditions were positively associated with multimorbidity, regardless of definition or threshold.

**Conclusions:**

Depending on definition and threshold, multimorbidity is either rare or endemic in working-age Australians. Irrespective of definition, musculoskeletal conditions are a near-ubiquitous feature of multimorbidity.

**Electronic supplementary material:**

The online version of this article (doi:10.1186/s13104-017-2376-4) contains supplementary material, which is available to authorized users.

## Background

Musculoskeletal conditions, such as arthritis, are highly prevalent throughout the world and create a substantial health burden for individuals, health systems and society due to their clinical features, fluctuating nature and inherent complexity of treatment regimens [1–4]. Musculoskeletal conditions are not exclusively a disease of old age, as almost one in five working-age people (aged 18–64 years) have a musculoskeletal condition [5–7]. For working-aged people, musculoskeletal conditions can have substantial employment and financial impacts [8, 9]. Furthermore, co-occurring conditions are increasingly prevalent amongst those with musculoskeletal conditions [5, 6, 10–14], and musculoskeletal conditions are common components of multimorbidity clusters [15, 16]. Multimorbidity increases health expenditure [17], usage of health care services, polypharmacy and mortality rates, and reduces functional status and quality of life [18–23]. Therefore, it is important to consider multimorbidity when treating people with musculoskeletal conditions [23]. Consequently, clinical and policy efforts to improve treatment of musculoskeletal conditions cannot be implemented without regard to the possible impacts of other conditions [12, 16–18, 24–26].

In addressing the issue of multiple co-occurring chronic conditions, a number of terms are used in the literature, including *multimorbidity* [11, 14], and *comorbidity* [6, 14, 27]. Clinically, ‘multimorbidity’ conceptualises and treats all coexisting conditions as one distinct entity within an individual, with all conditions having equal importance [21]. ‘Comorbidity’ focuses on a single index condition and considers additional conditions in relation to their causality (consequence or coincidence) with, or on, this index condition [21].

Epidemiologically, ‘multimorbidity’ applies when estimating the prevalence of co-occurring conditions among the wider population, and ‘comorbidity’ applies when estimating co-occurring conditions among populations with an index condition [28]. This distinction is important because estimates that include those without any disease at all result in a lower multimorbidity prevalence, compared to disease-specific subpopulations where all individuals have an index condition by definition [6, 14]. When estimating how related a particular condition and multimorbidity are, the most relevant comparison group is a population with other types chronic conditions. For example, when determining the association between musculoskeletal conditions and multimorbidity, those with *any condition other than a musculoskeletal condition* is the most relevant comparison group. This is because the presence of one condition increases risk of multimorbidity in disease-specific populations, due to shared life-style and biomedical disease risk factors, as well as medicines use [29] or induced diseases [30, 31], which increases the likelihood of developing another condition [32].

Despite its recognised importance (and cost), discussions of multimorbidity are hampered by inconsistent conceptual and operational definitions of what constitutes multimorbidity, making comparisons across studies problematic [16]. A fundamental difference is the nominal ‘multimorbidity threshold’ (i.e. the minimum number of conditions constituting multimorbidity). While the most common and intuitive threshold for multimorbidity is at least two conditions (the ‘2+’ threshold), recent research recommends that in conjunction with this, a threshold of at least three conditions (‘3+’ threshold) should also be used to provide a more reliable estimate of multimorbidity [33].

Alongside issues of threshold, study comparability is further limited by the number of variants of ‘operational definitions’ of multimorbidity, which stipulate the conditions counted in any pre-specified list used to estimate population levels of multimorbidity [33–36]. What is counted as a condition contributing to multimorbidity can be quite arbitrary. Unfortunately, there is no ‘gold standard’ as a reference point.

In addition to variations in inclusion of specific conditions that constitute multimorbidity, the level of condition abstraction varies. Studies inconsistently combine clinically distinct conditions into broad umbrella categories (e.g. ‘musculoskeletal conditions’ [11], ‘joint disease’ [37] or ‘arthritis’ [5–7, 15], known as ‘lumping’), while others focus on single conditions, sometimes to the exclusion of others (e.g. ‘osteoarthritis’ [38], divorced from all other musculoskeletal conditions, known as ‘splitting’). Lumping or splitting of conditions is potentially problematic for identifying patterns in multimorbidity, particularly that which includes musculoskeletal conditions given their clinically (and socially) heterogeneous nature.

Another inherent difficulty in comparing multimorbidity studies is variation in the estimates themselves, which can be attributed to differences in the population selected for study. Estimates of population prevalence of multimorbidity are typically based on populations attending particular services or GP clinics [11, 39]. Such populations will naturally be skewed towards older and/or less healthy people than within the wider population [40], potentially overestimating multimorbidity prevalence. Conversely, sample populations drawn from those currently employed in the workforce potentially skew towards those in better health, underestimating prevalence [15]. Reliable estimates of population prevalence of multimorbidity that include musculoskeletal conditions are particularly lacking and in an Australian context, where research is limited to the elderly and/or patients consulting their general practitioner, or to healthier, currently-employed workplace-based samples [5, 6, 11, 13, 15, 38, 41, 42].

To date, only one study has directly examined the influence of applying differing definitions of multimorbidity when estimating prevalence, and this study sampled from a general practice setting [39]. This direct comparison illustrates that the magnitude of multimorbidity prevalence varies significantly across the different nominal thresholds and operational definitions used [39]. However, the impact of thresholds and definitions has yet to been examined in the community-based population.

Due to these inconsistencies, it remains to be determined whether the prevalence of multimorbidity is uniform across different musculoskeletal populations, or if musculoskeletal conditions in general, or only particular subgroups of musculoskeletal conditions, are associated with increased prevalence of multimorbidity. Such information may help identify common groups of multimorbidities and therefore potential target populations for interventions aimed at ameliorating the increased burden associated with multimorbidity.

We sought to identify the influence of various definitions and thresholds on multimorbidity prevalence and association estimates. Specifically, this study estimates prevalence of, and association between, musculoskeletal and co-occurring conditions, and evaluates the implications of the following differences in multimorbidity definitions:

- Changing the minimum number (nominal threshold) of conditions that constitute multimorbidity (i.e. the 2+ and 3+ condition thresholds);
- Changing the range of chronic conditions included (operational definition) by examining three existing, pre-defined lists of conditions for operationalising multimorbidity, drawn from ‘survey-’ [43], ‘policy-’ [44] and ‘research-based’ [35] contexts; and
- Changing the level of abstraction of conditions considered musculoskeletal by lumping and splitting the musculoskeletal sample population.

We use data from the Australian Bureau of Statistics’ *National Health Survey 2007*–*08* [43], a representative population sample. To address a current gap in knowledge, we specifically focus on the data for the working-age population.

## Methods

### Data source

We used cross-sectional data from the Australian Bureau of Statistics’ (ABS) *National Health Survey 2007*–*08* (*NHS 2007*–*08*) [43]. The population survey is a nationally representative sample of 20,788 people from all Australian states and territories and across all age groups. One person aged 18 years and over and one child were randomly selected from 15,800 private households across Australia to be surveyed. Interviews, covering a wide range of self-reported personal health information, were completed by the ABS with an adult and, where relevant, a child (following parent or guardian consent). The overall response rate for the *NHS 2007*–*08* was 91%. The collection of NHS data and participant obligations and safeguards are governed by the *Census and Statistics Act 1905* (Cth). Under the Act, participants are required to provide the information requested. The ABS is obliged by the Act to maintain the privacy of all information provided. No information is released in a way that would enable an individual or household to be identified. Detailed information about sampling, survey design and questions asked is available elsewhere [43].

This study was granted exemption from ethics review by the La Trobe University Faculty of Health Sciences Faculty Ethics Review Committee due to negligible risk.

### Sample

For the purpose of this study, the sample population was defined as working-age survey respondents (18–64 years). The primary group of interest were those self-reporting chronic musculoskeletal conditions, considered as a heterogeneous lumped group (i.e. ‘musculoskeletal conditions’), as well as split in homogenous subgroupings (e.g. ‘osteoarthritis’) at the most basic level of abstraction available within the data collected. The homogenous musculoskeletal subgroups were: osteoarthritis; inflammatory arthritis; other arthritis or arthropathies; soft tissue disorders; back pain; gout; osteoporosis; and other musculoskeletal conditions (see Additional file 1: Table S1). We considered a musculoskeletal condition ‘chronic’ if it was: (1) current at the time of interview; and (2) reported as being present for six months or more.

### Multimorbidity nominal thresholds and operational definitions

Two nominal multimorbidity thresholds (i.e. minimum of 2+ and 3+ conditions) were compared in this analysis, alongside three different operational definitions (see Additional file 2: Table S2 for complete list of conditions included in each of the survey-, policy- and research-based definitions; see Additional file 3 for glossary of terms). Within Additional file 2: Table S2, columns 2, 4, and 6 detail the actual conditions included in each definition, while columns 3 and 5 detail the condition groups at the abstracted level:

- *Survey*-*based definition* An open-ended definition, inclusive of all long-term conditions reported by respondents as part of the *NHS 2007*–*08*. This definition includes the presence of all current diagnoses (illnesses, injuries or disabilities) self-reported as having been present for six months or more at the time of the interview, and were subsequently classified to 107 condition categories using ICD-10-AM (see Additional file 2: Table S2: column 2). Within this multimorbidity definition, injuries (such as fractures, amputations, nerve damage, and joint injury) and infections (such as HIV, hepatitis C, or tuberculosis) were included. These conditions were current and present for six months, which suggests they could be permanent or required prolonged rehabilitation or treatment.
- *Policy*-*based definition* This definition included the chronic health conditions identified as Australian National Health Priority Areas (NHPAs) based on high population prevalence and high impact on individuals and the health system [44]. These include: musculoskeletal conditions, diabetes, cancer, cardiovascular disease, asthma, chronic obstructive pulmonary disease, and mental health disorders (see Additional file 2: Table S2: columns 3 and 4). Although classified as NHPAs, obesity and injury prevention/control were excluded from this analysis. Obesity was excluded on the basis that there is disagreement that it is a disease [45, 46], and its ineligibility for Medicare-based coverage on its own [47]: furthermore, obesity data was available for only a sub-set of respondents. Injury prevention/control was not included as these are not diseases per se. Data on dementia was subsumed within an umbrella category of conditions included in the mental health disorder category (symptoms and signs involving cognition, perceptions, emotional state and behaviour), however, dementia is uncommon among the working-age population [48].
- *Research*-*based definition* The definition was based on a recent literature review by Diederichs et al. [35], which recommends inclusion of the following 11 conditions in multimorbidity studies: cancer, diabetes mellitus, depression, hypertension, myocardial infarction, chronic ischemic heart disease, heart arrhythmias, heart insufficiency, stroke, chronic obstructive pulmonary disease, and arthritis (see Additional file 2: Table S2: columns 5 and 6). This definition was modified slightly for this study by combining myocardial infarction and chronic ischemic heart disease, as it was not possible to separate the two in the available data. Notably, unlike the other two definitions, this definition excludes non-arthritis musculoskeletal conditions. As such, the presence of soft tissue disorders, back pain, osteoporosis, and other musculoskeletal conditions were not counted towards multimorbidity with this definition.

As above, for all definitions, the selected conditions were only included if they were chronic; that is, they were both current and present for six months or more.

### Analyses

Utilising the *NHS 2007*–*08*, confidentialised unit record file (CURF) data, weighted population prevalence and associations taking into account survey design, were estimated with the jack-knife method applying the replicate weights provided by the ABS [49]. Prevalence was estimated for each definition–threshold combination to determine the extent of multimorbidity among the working-age population with or without musculoskeletal conditions. Specifically, for each multimorbidity definition–threshold combination we estimated the:

1. Proportion of respondents with multimorbidity, from the total working-age population. This is represented by ^C^/_A_ in Fig. 1;

**Fig. 1:**
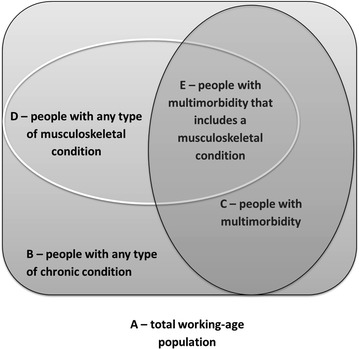
Illustration of broad populations of interest
2. Proportion of respondents with multimorbidity that includes at least one musculoskeletal condition, from the total working-age population. This is represented by ^*E*^/_*A*_ in Fig. 1;
3. Proportion of respondents with a musculoskeletal condition, from the multimorbidity subsample. This is represented by ^*E*^/_*C*_ in Fig. 1; and
4. Proportion of co-occurring conditions among the working-age population with any (lumped), and specific (split) musculoskeletal condition(s). This is represented by ^*E*^/_*D*_ in Fig. 1.

Finally, we used logistic regression to identify the odds of multimorbidity in persons with musculoskeletal conditions, compared with the odds of multimorbidity occurring in persons with non-musculoskeletal conditions, whilst taking into account any age and gender differences between each group. The reference group was the working-age population with any non-musculoskeletal conditions (i.e. B minus D in Fig. 1) included in the particular multimorbidity operational definition forming the analysis. As above, for each operational definition used, we compared estimates obtained when the minimum number of diagnoses (i.e. nominal threshold) that constitute multimorbidity/comorbidity varied (2+ and 3+ conditions) [33]. Odds ratios and confidence intervals were estimated using logistic regression, adjusting for age and gender. All statistical analyses were performed using Stata (release 10.1, College Station, TX, USA).

## Results

### Weighted population prevalence estimates

Of the 20,788 *NHS 2007*–*08* sample, 12,604 survey respondents were of working-age; of these, 4555 self-reported at least one current chronic musculoskeletal condition. Compared to those without musculoskeletal conditions, working-age people with musculoskeletal conditions were more likely to be older (43.7 vs 20.5%), born in Australia (74.7 vs 69.6%), and report the presence of three or more of the following disease risk factors of obesity; high blood pressure; osteoporosis; high cholesterol; high blood sugar; risky level of alcohol consumption; current smoker; insufficient vegetable intake; insufficient fruit intake; and sedentary lifestyle (42.5 vs 33.9%). However, they were less likely to live in a major city (65.1 vs 72.05%), work full-time (50.3 vs 58.9%) or possess an undergraduate degree (19.8 vs 24.2%) (Table 1).

**Table 1:** Demographic characteristics of working-age (18–64 years) respondents; and the subsets with and without musculoskeletal conditions (MSK)

Characteristics	Total % (n = 12,604)	With MSK % (n = 4555)	No MSK % (n = 8049)	P value
Age group				<0.01
18–34	37.1	21.5	45.0	
35–49	34.6	34.8	34.5	
50–64	28.3	43.7	20.5	
Gender				0.82
Female	50.1	50.3	50.0	
Place of birth				<0.01
Australia	71.3	74.7	69.6	
Household equivalised income quintile				<0.01
First quintile	9.6	13.6	7.5	
Second quintile	14.4	14.9	14.1	
Third quintile	19.3	19.6	19.2	
Fourth quintile	20.8	19.1	21.6	
Fifth quintile	20.6	17.9	22.0	
Not stated	15.3	14.8	15.6	
Education level				<0.01
Year 12 or less	43.5	43.3	43.6	
Diploma/certificate	33.8	36.9	32.2	
Bachelor or higher	22.8	19.8	24.2	
Hours worked				<0.01
No hours	22.3	28.9	19.0	
1–34 h	21.7	20.8	22.2	
35+ h	56.0	50.3	58.9	
Region of Australia				<0.01
Major cities	69.7	65.1	72.0	
Inner regional	19.9	23.1	18.3	
Other areas	10.4	11.8	9.7	
Household structure				<0.01
Couple and child(ren)	43.3	39.7	45.1	
Parent and child(ren)	8.3	7.9	8.5	
Couple only	23.0	27.5	20.8	
Single person	10.9	13.2	9.7	
Other	14.5	11.7	15.9	
Number of disease risk factors^a^				<0.01
None	6.1	5.2	6.6	
1	24.5	22.6	25.5	
2	32.6	29.8	34.0	
3 or more	36.8	42.5	33.9	

All estimates adjusted for survey design

^a^Disease risk factors: obesity; high blood pressure; osteoporosis; high cholesterol; high blood sugar; risky level of alcohol consumption; current smoker; insufficient vegetable intake; insufficient fruit intake; and sedentary lifestyle

### Multimorbidity in the total working-age population (^*C*^/_*A*_; Fig. 2)

Using the survey-based definition, the proportion of the Australian working-age population (n = 12,604) considered multimorbid was 61.5% (95% confidence interval [CI] 60.3–63.7%) at the 2+ condition threshold (Table 2). Using the policy-based definition, multimorbidity prevalence was 15.3% (95% CI 14.3–16.2%). Multimorbidity prevalence was lowest with the research-based definition: 7.9% (95% CI 7.4–8.5%). Notably, 1034 participants with non-arthritis musculoskeletal conditions are excluded from the multimorbidity count with this research-based definition. Using the 3+ condition threshold, multimorbidity prevalence rates decreased to 41.9% (95% CI 40.6–43.1%); 4.2% (95% CI 3.7–4.7%); and 2.1% (95% CI 1.8–2.5%) for the survey-, policy- and research-based definitions respectively (Table 2).

**Fig. 2:**
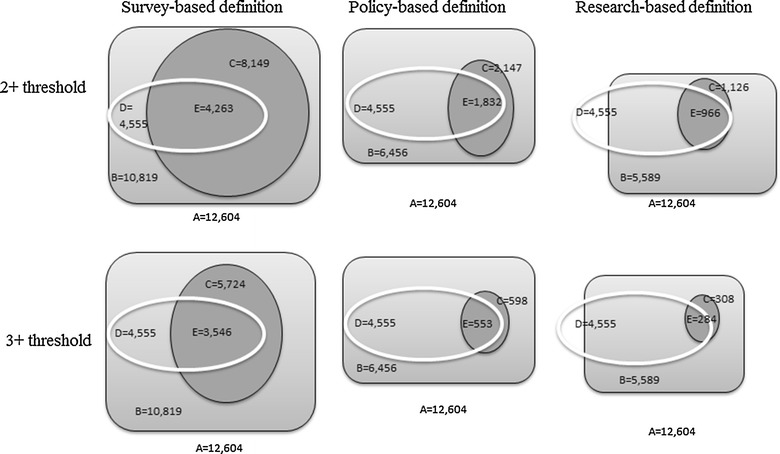
Overlap between populations with musculoskeletal conditions and multimorbidity as defined by each definition and threshold. **a** Total working-age sample population; **b** sub-sample with at least one condition; **c** sub-sample with multimorbidity; **d** sub-sample with any musculoskeletal condition; **e** musculoskeletal sub-sample considered multimorbid

**Table 2:** Working-age population prevalence of (1) multimorbidity, (2) any musculoskeletal condition (MSK) and multimorbidity, and (3) MSK among those with multimorbidity

Population description	Prevalence % (95% CIs)^a^			
	Condition threshold	Survey-based definition^b^	Policy-based definition^c^	Research-based definition^d^
1. Multimorbidity among the working-age population (i.e. ^C^/_A_; Fig. 2) (n = 12,604)	2+	61.5 (60.3, 62.7)	15.3 (14.3, 16.2)	7.9 (7.4, 8.5)
	3+	41.9 (40.6, 43.1)	4.2 (3.7, 4.7)	2.1 (1.8, 2.5)
2. Multimorbidity that includes at least one MSK among the working-age population (i.e. ^E^/_A_; Fig. 2) (n = 12,604)	2+	31.1 (29.9, 32.3)	12.9 (12.1, 13.7)	6.8 (6.3, 7.4)
	3+	25.4 (24.3, 26.5)	3.8 (3.4, 4.3)	2.0 (1.6, 2.3)
3. MSK among the working-age subsample with multimorbidity (i.e. ^E^/_C_; Fig. 2) (n = varies for each multimorbidity definition)	2+	50.6 (49.1, 52.1)	84.8 (83.0, 86.5)	86.0 (83.4, 88.5)
	3+	60.7 (58.9, 62.5)	91.8 (88.4, 95.2)	93.5 (90.4, 96.5)

^a^All prevalence estimates are based on the total Australian working-age population (ages 18-64 years) by taking the NHS survey design weightings into account, unless otherwise specified

^b^Survey-based: multimorbidity defined as including any two or more of the possible conditions measured in the Australian National Health Survey that were reported as being present for 6 months or more

^c^Policy-based: multimorbidity defined as including two or more of any of the restricted classes of morbidities based on Australian National Health Priority Areas being present for 6 months or more: musculoskeletal conditions, diabetes, cancer, cardiovascular disease, asthma, chronic obstructive pulmonary disease, mental health

^d^Research-based: 11 specific conditions being present for 6 months or more, based on a literature review conducted by Diederichs et al. [35], cancer, diabetes mellitus, depression, hypertension, myocardial infarction, chronic ischemic heart disease, heart arrhythmias, heart insufficiency, stroke, chronic obstructive pulmonary disease, and arthritis

### Working-age population with multimorbidity that includes a musculoskeletal condition (^*E*^/_*A*_; Fig. 2)

Within the working-age population, the weighted population prevalence of multimorbidity involving a musculoskeletal condition varied dramatically between definitions. These estimates ranged between 31.1% (95% CI 29.9–32.3%) with the survey definition with 2+ threshold, to as few as 2.0% (95% CI 1.6–2.3%) with the research definition with 3+ threshold.

### Lumped musculoskeletal conditions among working-age population with multimorbidity (^*E*^/_*C*_; Fig. 2)

Regardless of threshold, using either the policy- or research-based definition, the vast majority of those with multimorbidity have at least one musculoskeletal condition [84.8% (95% CI 83.0–86.5%) and 86.0% (95% CI 83.4–88.5%)] (see Table 2). A lower proportion [50.6% (95% CI 49.1–52.1%)] of those captured by the survey-based definition have multimorbidity that involves musculoskeletal conditions. When a 3+ condition threshold was used, the proportion of those with a musculoskeletal condition within the populations with survey-, policy- and research-defined multimorbidity rose to 60.7% (95% CI 58.9–62.5%); 91.8% (95% CI 88.4–95.2%) and 93.5% (95% CI 90.4–96.5%) respectively.

### Comorbidities among the working-age population with any lumped musculoskeletal condition (^*E*^/_*D*_; Fig. 2)

Among working-age people with at least one musculoskeletal condition (*n* = 4555), the prevalence of co-occurring conditions was higher than in the working-age population without musculoskeletal conditions; however, prevalence varied widely with the different definitions and thresholds used (Table 3). Among working-age people with musculoskeletal conditions, the proportion at the 2+ threshold (where one condition was a musculoskeletal condition) was 92.2% (95% CI 90.9–93.3%; survey-based definition); 38.3% (95% CI 36.5–40.1%; policy-based) and 20.2% (95% CI 18.9–21.6%; research-based). Using a 3+ threshold (where one condition is a musculoskeletal condition), prevalence of comorbidity was 75.4% (95% CI 73.6–77.0%); 11.4% (95% CI 10.1–12.8%); 5.9% (95% CI 4.9–7.0%) for the same definitions, respectively.

**Table 3:** Working-age population prevalence of multimorbidity among those with musculoskeletal conditions (MSK) (lumped and split into subgroups)

	Multimorbidity prevalence % (95% CIs)		
	Survey-based definition^a^	Policy-based definition^a^	Research-based definition^a^
*1. Prevalence of* *two* *or more co*-*occurring conditions (2*+ *threshold)* ^b^			
**Any MSK (n** **=** **4555)**	**92.2 (90.9, 93.3)**	**38.3 (36.5, 40.1)**	**20.2 (18.9, 21.6)**
Osteoarthritis	98.5 (97.3, 99.2)	46.1 (41.3, 51.1)	40.4 (36.2, 44.8)
Inflammatory arthritis	98.2 (90.5, 99.7)	53.1 (45.2, 61.0)	45.3 (37.3, 53.5)
Other arthritis	95.0 (93.2, 96.4)	41.9 (38.1, 45.8)	27.2 (23.4, 31.2)
Gout	90.9 (87.0, 93.7)	37.8 (32.8, 43.1)	33.9 (29.1, 39.0)
Other musculoskeletal	95.6 (88.0, 98.5)	41.4 (28.0, 56.3)	22.7 (12.4, 37.8)
Back pain	91.7 (90.0, 93.2)	39.8 (37.6, 42.0)	15.8 (14.0, 17.8)
Soft tissue conditions	95.9 (91.3, 98.1)	46.9 (40.3, 53.6)	25.4 (20.1, 31.5)
Osteoporosis	99.0 (96.0, 99.8)	50.4 (42.1, 58.7)	27.4 (20.3, 36.0)
*2. Prevalence of* *three* *or more co*-*occurring conditions (3*+ *threshold)*			
**Any MSK**	**75.4 (73.6, 77.0)**	**11.4 (10.1, 12.8)**	**5.9 (4.9, 7.0)**
Osteoarthritis	90.7 (88.0, 92.9)	16.4 (13.2, 20.2)	11.3 (8.6, 14.7)
Inflammatory arthritis	88.0 (79.7, 93.2)	23.0 (17.1, 30.2)	14.7 (9.5, 22.0)
Other arthritis	81.8 (78.5, 84.7)	14.7 (12.0, 17.8)	8.5 (5.8, 12.3)
Gout	77.3 (72.4, 81.6)	10.6 (8.1, 13.7)	10.4 (7.8, 13.7)
Other musculoskeletal	79.0 (66.8, 87.6)	11.8 (6.0, 22.0)	6.5 (2.9, 13.8)
Back pain	76.0 (73.4, 78.3)	11.7 (10.0, 13.7)	5.1 (4.2, 6.4)
Soft tissue conditions	82.9 (76.4, 87.9)	18.07 (13.5, 23.8)	7.6 (5.0, 11.4)
Osteoporosis	89.1 (81.8, 93.7)	17.3 (13.1, 22.5)	9.0 (5.1, 15.5)

^a^See footnote of Table 2 for details of each multimorbidity definition

^b^All prevalence estimates representative of the whole of the Australian working-age population (ages 18–64 years) by taking the NHS survey design weightings into account, unless otherwise specified

### Comorbidities among the working-age population with specific split musculoskeletal conditions (sub-groups within ^*E*^/_*D*_; Fig. 2)

For the survey-based definition, when the level of abstraction is split into specific musculoskeletal subgroups, the subgroups with highest multimorbidity prevalence with 2+ threshold were osteoporosis (99.0%); osteoarthritis (98.5%) and inflammatory arthritis (98.2%), while the lowest prevalence rates were observed for gout (90.9%) and back pain (91.7%) (Table 3). With the policy-based definition, the musculoskeletal subgroups with highest prevalence with 2+ threshold were inflammatory arthritis (53.1%), osteoporosis (50.4%), soft tissue conditions (46.9%) and osteoarthritis (46.1%) and lowest prevalence again were with gout and back pain (37.8 and 39.8%, respectively). Prevalence (with 2+ threshold) was also highest among those with inflammatory arthritis (45.3%); osteoarthritis (40.4%) and gout (33.9%) using the research-based definition and lowest again for those with back pain (15.8%) and this time, those with other musculoskeletal conditions not elsewhere described (22.7%). Increasing to the 3+ condition threshold did not substantially alter the musculoskeletal subgroups with highest and lowest prevalence of multimorbidity; however, rates were reduced slightly.

### Associations between musculoskeletal conditions and multimorbidity

After adjustment for age and gender, the odds of multimorbidity were greater in respondents with musculoskeletal conditions than those with any of the non-musculoskeletal conditions included in each definition of multimorbidity assessed. However, there were variations in the strength of these associations, with the strongest associations seen for the survey-based definition [adjusted Odds Ratio (aOR) 6.7 (95% CI 5.6–8.1)]; and weakest for the policy-based definition [aOR 1.5 (95% CI 1.2–2.0)]. As such, the strength of the associations seems related to the prevalence produced by the definition; strongest associations are seen for definitions producing the greatest prevalence of multimorbidity. The magnitude in the difference between these associations produced by each definition was greater with the two condition multimorbidity threshold (as opposed to the three condition threshold where the confidence intervals of the effect estimates often overlapped). This pattern of results also applied when examining the specific musculoskeletal conditions. For example, the odds of survey-based multimorbidity among persons with osteoarthritis was 30.0 times that of persons with any other non-musculoskeletal condition comprising the survey definition (95% CI 15.2–59.2); while the comparable associations for the policy and research definitions were aOR 3.7 (95% CI 2.7–4.9) and aOR 3.3 (95% CI 2.5–4.5) (Table 4).

**Table 4:** Adjusted associations between multimorbidity and musculoskeletal conditions

	Multimorbidity OR (95% CI)^a, b^		
	Survey-based definition^c^	Policy-based definition^c^	Research-based definition^c^
*1.* *Two* *or more co*-*occurring conditions (2*+ *threshold)*			
**Musculoskeletal**	**6.7 (5.6, 8.1)**	**3.0 (2.6, 3.5)**	**1.5 (1.2, 2.0)**
Osteoarthritis	30.0 (15.2, 59.2)	3.7 (2.7, 4.9)	3.3 (2.5, 4.5)
Inflammatory arthritis	26.1 (0.7, 1035.2)	5.0 (3.3, 7.7)	4.4 (2.9, 6.7)
Other arthritis	10.5 (7.4, 15.1)	3.4 (2.8, 4.2)	2.1 (1.5, 2.9)
Gout	5.7 (3.7, 8.7)	2.8 (2.1, 3.7)	2.7 (2.0, 3.8)
Other musculoskeletal	13.3 (3.9, 45.7)	3.5 (1.9, 6.6)	1.8 (0.8, 3.9)
Back pain	6.9 (5.5, 8.6)	3.3 (2.8, 4.0)	1.3 (1.0, 1.6)
Soft tissue conditions	12.3 (5.4, 28.1)	4.0 (2.9, 5.4)	1.9 (1.3, 2.8)
Osteoporosis	40.4 (5.6, 291.1)	4.3 (3.0, 6.2)	1.9 (1.1, 3.1)
*2.* *Three* *or more co*-*occurring conditions (3*+ *threshold)*			
**Musculoskeletal**	**5.6 (4.9, 6.3)**	**4.7 (2.9, 7.5)**	**3.6 (2.1, 6.0)**
Osteoarthritis	14.2 (10.5, 19.3)	7.2 (3.7, 13.8)	5.7 (3.4, 9.4)
Inflammatory arthritis	11.7 (6.0, 22.7)	11.2 (4.9, 25.9)	8.0 (3.8, 17.0)
Other arthritis	8.0 (6.3, 10.1)	6.1 (3.4, 10.9)	4.9 (2.4, 10.3)
Gout	6.3 (4.7, 8.3)	3.8 (2.0, 6.9)	5.1 (2.9, 9.0)
Other musculoskeletal	7.3 (4.0, 13.2)	4.8 (1.7, 13.4)	4.3 (1.7, 11.3)
Back pain	6.3 (5.4, 7.4)	5.1 (3.1, 8.3)	3.7 (2.2, 6.2)
Soft tissue conditions	8.3 (5.7, 12.1)	7.2 (4.0, 13.2)	4.4 (2.0, 9.9)
Osteoporosis	10.7 (5.7, 20.0)	6.8 (3.0, 15.2)	4.9 (1.9, 12.9)

^a^ Association estimates based on comparison with respondents with any of the non-musculoskeletal conditions in each definition

^b^ Adjusted for age and gender

^c^ See footnote of Table 2 for details definitions of multimorbidity used in analyses

## Discussion

We found that within the Australian working-age population, prevalence estimates for multimorbidity varied greatly from 61.5% in the unrestricted survey-based definition (2+ threshold) to 15.3% (policy) and 7.9% (research) definitions, despite each purporting to measure the same phenomenon.

This finding is consistent with other research into the impact of multimorbidity definitions [39, 50] and thresholds [39, 51] although Fortin et al. [50] examined prevalence in different source populations making it difficult to determine if the differences in prevalence estimate was due to the different definitions or the underlying burden of disease in the populations assessed. Our estimates from a nationally-representative population sample are similar to those reported in a study of an Australian population [39, 51] sampled from general practice, with conditions identified via doctor diagnosis and different criteria for chronicity.

Our study comprehensively estimates the extent of multimorbidity among the musculoskeletal population—a population with clinical and policy relevance [44]. Regardless of the definition or threshold, musculoskeletal conditions are a common component of multimorbidity. Most working-age adults who met the policy and research definition of multimorbid had a musculoskeletal condition. This is an important finding because the inclusion of musculoskeletal conditions within multimorbidity research has been inconsistent.

Musculoskeletal conditions are a clinically heterogeneous group and it is plausible that the prevalence of multimorbidity might vary for sub-types of musculoskeletal conditions with each definition. The subgroups with the highest prevalence of multimorbidity were typically osteoporosis, osteoarthritis and inflammatory arthritis.

There is a lack of conceptual clarity for multimorbidity as a construct, as evidenced by the large number of multimorbidity definitions already in use and the wide-ranging estimates of prevalence they generate. Despite each definition investigated here purporting to measure multimorbidity prevalence estimates varied greatly, demonstrating the elastic nature of multimorbidity as a concept. Consistency of definition is critical for comparing the burden of multimorbidity between and within populations, and changes over time. Despite the potential benefits of a consistent definition of multimorbidity, it may actually require more than a single definition, so long as the implications of such differences are understood. This will enable definitions to be fit for purpose, relevant to specific contexts and allow consideration of the relevant multimorbidity features.

There are advantages and disadvantages for using each of the definitions examined here. The survey-based definition includes all chronic conditions self-reported by respondents and is therefore a comprehensive indication of what the respondents themselves consider as being co-occurring conditions. However, some may not be generally considered medical conditions (e.g. astigmatism) or may be conditions, that while chronic, may in some cases manifest themselves episodically or infrequently (e.g. migraines or asthma) and/or may not require medications to treat or manage (e.g. certain injuries). Nonetheless, these conditions may be important for understanding multimorbidity [52]. However, the extremely high prevalence rates derived by the survey definition over emphasises the problem because it captures almost everyone and everything. This renders the utility of the survey definition as an indicator questionable, as multimorbidity reaches endemic proportions, but more often than not will have questionable clinical relevance. However, the substantially higher prevalence derived from the survey definition highlights that the policy and research definitions do not capture the breadth of multimorbidity experienced within the working-age population. Previous research suggests the expression of multimorbidity is different in this younger population [53]. Further research may need to be conducted to identify other clinically important (such as epilepsy) and common chronic conditions specific to working-age adults with multimorbidity, which are currently not captured by either the policy or research definitions.

The policy definition counts conditions identified as NHPAs for Australia [44]. This restricted inclusion of conditions, results in lower prevalence estimates, and more clinically relevant multimorbidity than that derived by the survey definition. However, the policy definition uses a level of highly-abstracted diagnosis. For example, a person with depression and comorbid anxiety would be attributed with just one condition (‘mental health’) using the policy definition. This creates a case of severe lumping and under-estimation, particularly for musculoskeletal and mental health conditions. However, it may be appropriate to group related conditions when self-reporting respondents may not be expected always to be familiar with medical terminology. For example, some individuals may not recognise their condition as specifically ‘ischemic heart disease’ or ‘myocardial infarction’, however, they may nominate they have these conditions when an umbrella term is used, such as ‘heart diseases’ [35]. As *NHS 2007*–*08* data relied on self-report, the abstracted (lumped level) of conditions for the policy definition is arguably more appropriate.

The research definition is restricted to conditions that are burdensome to individuals and is informed by a literature review of research [35]. However, in contrast to the policy definition, the research definition appears arbitrary about the range and level of abstraction of conditions included, which may lead to both under and over -estimation of multimorbidity. For example, although depression is included in the research definition, all other mental health conditions are excluded, potentially contributing to under-estimation of multimorbidity. Similarly, although all arthritis conditions are included as an umbrella category, osteoporosis, back pain, soft tissue and other musculoskeletal conditions are excluded. In the context of this paper, the omission of non-arthritis musculoskeletal conditions is problematic, as the specific index condition (a musculoskeletal condition) will not be uniformly counted (or not counted), and there for result in bias in both prevalence estimates and measures of association. As a consequence lower multimorbidity prevalence rates are observed within these non-arthritis musculoskeletal subgroups. For example, those with an excluded musculoskeletal condition, such as osteoporosis, would actually have (at least) three conditions to be considered multimorbid using the 2+ threshold with this research definition. Furthermore, the research definition is highly skewed to heart and cardiovascular diseases; with six specific forms of cardiovascular disease each counted individually towards multimorbidity, potentially contributing to over-estimation. The definition we use here is slightly modified from that recommended by Diederichs et al. [35], due to myocardial infarction and chronic ischemic heart disease being combined within the *NHS 2007*–*08* data, so it counts five rather than six of these cardiovascular diseases.

As with previous studies, we have shown that the nominal threshold of the number of conditions contributing to multimorbidity matters when estimating prevalence and association, i.e. there is lower prevalence with increased threshold [39, 51]. There is a close relationship between prevalence estimates from a 2+ and 3+ thresholds of multimorbidity operationalised with simple condition-counts [54]. Where the prevalence estimate for a 2+ threshold is known, the 3+ threshold can be estimated and vice versa [54]. However, knowing that this relationship exists does not guide the appropriate nominal threshold to use with a particular operational definition of multimorbidity. It has been suggested that the 3+ threshold should be called ‘complex morbidity’ (defined as the co-occurrence of three or more chronic conditions affecting three or more different body systems within one person without defining an index chronic condition) [39]. In our study, the presence of three or more conditions when using the research and policy definitions may be suggestive of ‘complex morbidity’. In contrast, due to the inclusive nature of the survey definition, it would seem unlikely that the 3+ threshold is indicative of ‘complex multimorbidity’. Rather, with the 3+ threshold, multimorbidity with the survey definition resembles multimorbidity derived from the research or policy definition with the 2+ threshold. Therefore, we suggest that with inclusive (open list) definitions like the survey-definition the 3+ threshold may be more appropriate than the 2+ threshold, particularly when comparing rates with studies using restrictive operational definitions such as the research or policy definition. Within multimorbidity operational definitions the inclusion of obesity, hypertension and hyperlipidaemia is inconsistent, reflecting broader debate around the status of these conditions that are sometimes considered modifiable risk factors for diseases or diseases in their own right [45–47]. We could not include obesity, although it is an Australian NHPA [46], because consistent data for BMI calculations was not collected. Further research is needed to better understand the appropriate threshold to use when definitions count these highly prevalent conditions towards multimorbidity.

Strengths of this study are use of Australian data that is population-based, community dwelling and of working aged adults. This avoids the limitations of clinical, convenience or opportunistic samples and fills the knowledge gap associated with sampling elderly populations or sampling specific forms of arthritis populations [5, 6, 11, 15, 38, 41, 42]. Furthermore, by conducting exploratory analyses within the same population we are able to identify the variations in estimates attributable to the operational definitions and nominal thresholds used and not to differences in geographical setting, recruitment and data collection methods. However, this analysis also has a number of limitations. As the sample was drawn from community dwelling populations and did not include people living in care facilities or hospitalised patients a selection bias towards sampling healthier respondents is plausible. Furthermore, as the data is based on self-reported conditions, this information could be subject to recall bias. None of the definitions were weighted according to the severity of condition. While a number of operational definitions weight condition severity [55–60], there is little evidence that they are more effective in predicting outcomes such as mortality or health care utilisation compared to the simpler disease counts [34]. Additionally, although overall ratings of quality of life and pain were reported by each individual surveyed within the *NHS 2007*–*08*, it is not possible to apply a weighting to a specific condition. Therefore, in the context of multiple chronic conditions, it is appropriate that we used simple disease counts to estimate population prevalence of multimorbidity.

## Conclusion

We identified that among the Australian community dwelling, working-age population, depending on threshold and definition used, multimorbidity is either a rare or endemic phenomenon. Regardless of definition, musculoskeletal conditions are a common component of multimorbidity, raising uncertainty for prevalence estimates which are limited to some forms of arthritis. There is a need to better understand multimorbidity that includes musculoskeletal conditions, in order to estimate burden to individuals and to prioritise prevention and treatment efforts. A next step includes determining forms of musculoskeletal conditions for which the health and healthcare burden are exacerbated in the presence of multimorbidity.

## Additional files

**Additional file 1.** Musculoskeletal conditions (lumped together and split into homogenous subgroups) outlined by category used in analysis, source and description of variable within the National Health Survey data (NHS 2007–08).

**Additional file 2.** Complete list of conditions included (and level of abstraction) for survey-, policy- and research-based definitions mapped to the National Health Survey (NHS 2007–08) data (description of source variable and CURF code).

**Additional file 3.** Glossary of terms.

## Abbreviations

ABS: Australian Bureau of Statistics
*NHS 2007*–*08*: National Health Survey 2007–08
ICD-10-AM: International Statistical Classification of Diseases and Related Health Problems, Tenth Revision, Australian Modification
NHPA: National Health Priority Areas
CURF: confidentialised unit record file
BMI: body mass index
