# Supplementary material for: Cross-sectional examination of musculoskeletal conditions and multimorbidity: influence of different thresholds and definitions on prevalence and association estimates
Source: BMC Res Notes. 2017 Jan 18;10:51. doi: 10.1186/s13104-017-2376-4 (PMC5242059; doi:10.1186/s13104-017-2376-4)
Supplement: Supplementary file 1 — Additional file 1. Musculoskeletal conditions (lumped together and split into homogenous subgroups) outlined by category used in analysis, source and description of variable within the National Health Survey data (NHS 2007–08). [file 13104_2017_2376_MOESM1_ESM.docx]

**Table S1: Musculoskeletal sample population (lumped together and split into homogenous subgroups) outlined by category used in analysis, source and description of variable within the National Health Survey data (NHS07-08)**

| **Musculoskeletal conditions split subgroups** | **Corresponds with ABS NHS 07-08 variable (Confidentialised Unit Record File (CURF) code)** |
| --- | --- |
| **Osteoarthritis** | Arthritis – osteoarthritis (23421)  Osteoarthritis (this code is auto coded when the respondent selected Osteoarthritis from the questionnaire list. This accounted for the majority (97%) of answers from respondents with osteoarthritis; other responses included degeneration of specific joint; knee osteoarthritis; and hip osteoarthritis) |
| **Inflammatory arthritis** | Arthritis – rheumatoid (23445)  Rheumatoid arthritis (this code is auto coded when the respondent picks Rheumatoid arthritis from the pick list in the questionnaire. This accounts for the majority of answers from respondents with rheumatoid arthritis - 95%; other responses included rheumatoid arthritis: autoimmune; ankylosing spondylitis; arthritis inflammatory) |
| **Other arthropathies or arthritis other type unknown** | Arthritis - other/type unknown (23422)  974 - Arthritis - type unknown (this code is auto coded when the respondent picks Arthritis - Type unknown from the pick list in the questionnaire. This accounts for the majority of answers from respondents with arthritis -type unknown - 90%) |
|  | Other arthropathies (23139) |
| **Soft tissue disorders** | Other soft tissue disorders (23140) |
|  | Rheumatism (23216) |
| **Back pain** | Back pain/problems (23406) |
|  | Sciatica (23425) |
|  | Disc disorders (23171) |
|  | Curvature of the spine (23242) |
| **Gout** | Gout (23692) |
| **Osteoporosis** | Osteoporosis (23455) |
| **Other Musculoskeletal conditions** | Symptoms, signs involving nervous and musculoskeletal system (23137) |
|  | Other diseases musculoskeletal system and connective tissue (23173) |
