# Supplementary material for: Cross-sectional examination of musculoskeletal conditions and multimorbidity: influence of different thresholds and definitions on prevalence and association estimates
Source: BMC Res Notes. 2017 Jan 18;10:51. doi: 10.1186/s13104-017-2376-4 (PMC5242059; doi:10.1186/s13104-017-2376-4)
Supplement: Supplementary file 2 — Additional file 2. Complete list of conditions included (and level of abstraction) for survey-, policy- and research-based definitions mapped to the National Health Survey (NHS 2007–08) data (description of source variable and CURF code). [file 13104_2017_2376_MOESM2_ESM.docx]

**Table S2: Complete list of condition inclusion (and level of abstraction) for survey-, policy- and research-based definition mapped to the National Health Survey data (description of source variable and CURF code)**

| **Basic level of abstraction/** **ICD-10-AM classification of chronic conditions available in the National Health Survey Data 2007-8 (CURF code)** | **Survey-based definition: all 107 conditions included** | **Policy-based definition level of abstraction –restricted to 7 condition groups** | **Policy-based definition condition inclusion** | **Research- based definition level of abstraction – restricted to 10 conditions groups** | **Research-based definition condition inclusion** |
| --- | --- | --- | --- | --- | --- |
| Certain infectious and parasitic diseases (11020) | √ |  | X |  | X |
| Neoplasms -skin (12041) | √ | Cancer | √ | Cancer | √ |
| Neoplasms - Other and site unknown (12070) | √ |  | √ |  | √ |
| Benign neoplasms and neoplasms of uncertain nature (12086) | √ |  | X |  | X |
| Diseases of blood and blood forming organs –Anaemias (13076) | √ |  | X |  | X |
| Diseases of blood and blood forming organs - Other (13064) | √ |  | X |  | X |
| Endocrine, nutritional and metabolic diseases -Disorders of thyroid gland (14673) | √ |  | X |  | X |
| Endocrine, nutritional and metabolic diseases -Diabetes mellitus -Type 1 (14688) | √ | Diabetes mellitus | √ | Diabetes Mellitus | √ |
| Endocrine, nutritional and metabolic diseases -Diabetes mellitus -Type 2 (14689) | √ |  | √ |  | √ |
| Endocrine, nutritional and metabolic diseases -Diabetes mellitus -Type unknown (14690) | √ |  | √ |  | √ |
| Endocrine, nutritional and metabolic diseases -High sugar levels in blood/urine (14691) | √ |  | X |  | X |
| Endocrine, nutritional and metabolic diseases -High cholesterol (14693) | √ |  | X |  | X |
| Endocrine, nutritional and metabolic diseases -Other (14055) | √ |  | X |  | X |
| Mental and behavioural problems -Alcohol and drug problems (15526) | √ | Mental Health | √ |  | X |
| Mental and behavioural problems -Mood (affective) problems- Feeling depressed (15515) | √ |  | √ | Depression | √ |
| Mental and behavioural problems - Mood (affective) disorders- Other (15541) | √ |  | √ |  | X |
| Other mental and behavioural problems -Anxiety related problems (15089) | √ |  | √ |  | X |
| Other mental and behavioural problems -Problems of psychological development (15534) | √ |  | √ |  | X |
| Other mental and behavioural problems -Behavioural and emotional problems with usual onset in childhood/adolescence (15520) | √ |  | √ |  | X |
| Other mental and behavioural problems- not elsewhere classified (15264) | √ |  | √ |  | X |
| Symptoms signs involving cognition perceptions emotional state and behaviour (15007) | √ |  | √ |  | X |
| Diseases of nervous system –Epilepsy (16505) | √ |  | X |  | X |
| Diseases of nervous system –Migraine (16506) | √ |  | X |  | X |
| Diseases of nervous system -Other (16112) | √ |  | X |  | X |
| Diseases of eye and adnexa –Cataract (17331) | √ |  | X |  | X |
| Diseases of eye and adnexa –Glaucoma (17332) | √ |  | X |  | X |
| Disorders of choroid and retina -Macular degeneration (17328) | √ |  | X |  | X |
| Other disorders of choroid and retina (17326) | √ |  | X |  | X |
| Astigmatism (17103) | √ |  | X |  | X |
| Presbyopia (17036) | √ |  | X |  | X |
| Short sight/myopia (17105) | √ |  | X |  | X |
| Long sight/hyperopia (17640) | √ |  | X |  | X |
| Other disorders of ocular muscles, binocular (17107) | √ |  | X |  | X |
| Complete or partial blindness (one or both eyes) (17093) | √ |  | X |  | X |
| Other visual disturbances or loss of vision (17305) | √ |  | X |  | X |
| Other diseases of eye and adnexa - Colour blind (17106) | √ |  | X |  | X |
| Other diseases of eye and adnexa- not elsewhere classified (17119) | √ |  | X |  | X |
| Diseases of ear and mastoid - Complete or partial deafness and hearing loss (18108) | √ |  | X |  | X |
| Diseases of middle ear and mastoid process - Otitis media (18346) | √ |  | X |  | X |
| Other diseases of middle ear and mastoid (18348) | √ |  | X |  | X |
| Diseases of inner ear (18111) | √ |  | X |  | X |
| Tinnitus (18967) | √ |  | X |  | X |
| Other diseases of the ear (18134) | √ |  | X |  | X |
| Diseases of circulatory system -Hypertensive disease (19392) | √ |  | X | Hypertension | √ |
| Ischaemic heart diseases –Angina (19117) | √ | Cardio-vascular disease | √ | Myocardial infarction/ chronic ischemic heart disease | √ |
| Other Ischaemic heart diseases (19382) | √ |  | √ |  | √ |
| Tachycardia (19365) | √ |  | √ | Heart arrhythmia | √ |
| Cerebrovascular diseases (19396) | √ |  | √ | Stroke | √ |
| Oedema and heart failure (19135) | √ |  | √ | Heart insufficiency | √ |
| Diseases of arteries, arterioles and capillaries (19114) | √ |  | √ |  | X |
| Diseases of veins, lymphatic vessels etc –Haemorrhoids (19403) | √ |  | X |  | X |
| Diseases of veins, lymphatic vessels etc -Varicose veins (19402) | √ |  | X |  | X |
| Other diseases of veins, lymphatic vessels (19067) | √ |  | X |  | X |
| Other diseases of circulatory system -Low blood pressure (19116) | √ |  | X |  | X |
| Other diseases of circulatory system (19377) | √ |  | √ |  | X |
| Symptoms, signs involving circulatory system - Cardiac murmurs and cardiac sounds (19389) | √ |  | √ |  | X |
| Other signs, symptoms involving circulatory system (19362) | √ |  | √ |  | X |
| Chronic lower respiratory diseases –Bronchitis (20583) | √ | Chronic obstructive pulmonary disease | √ | Chronic obstructive pulmonary disease | √ |
| Chronic lower respiratory diseases –Emphysema (20596) | √ |  | √ |  | √ |
| Chronic lower respiratory diseases –Asthma (20597) | √ | Asthma | √ |  | X |
| Other diseases of respiratory system -Hayfever and allergic rhinitis (20215) | √ |  | X |  | X |
| Other diseases of respiratory system -Chronic sinusitis (20580) | √ |  | X |  | X |
| All other diseases of respiratory system (20120) | √ |  | X |  | X |
| Symptoms, signs involving respiratory system (20009) | √ |  | X |  | X |
| Diseases of the oesophagus (21285) | √ |  | X |  | X |
| Stomach/duodenal/gastrointestinal ulcer (21287) | √ |  | X |  | X |
| Other diseases of the oesophagus, stomach and duodenum (21129) | √ |  | X |  | X |
| Hernia (21291) | √ |  | X |  | X |
| Other diseases of the intestines (21098) | √ |  | X |  | X |
| Gallstones (21486) | √ |  | X |  | X |
| All other diseases of the digestive system (21131) | √ |  | X |  | X |
| Symptoms, signs involving digestive system (21096) | √ |  | X |  | X |
| Dermatitis and eczema (22099) | √ |  | X |  | X |
| Psoriasis (22647) | √ |  | X |  | X |
| Other diseases of skin and subcutaneous tissue (22025) | √ |  | X |  | X |
| Symptoms, signs involving skin and subcutaneous tissue (22174) | √ |  | X |  | X |
| Arthropathies- Gout (23692) | √ | Musculo-skeletal conditions | **√** | Arthritis | **√** |
| Arthritis – Rheumatoid (23445) | √ |  | **√** |  | **√** |
| Arthritis – Osteoarthritis (23421) | √ |  | **√** |  | **√** |
| Arthritis - Other and type unknown (23422) | √ |  | **√** |  | **√** |
| Other arthropathies (23139) | √ |  | **√** |  | **√** |
| Soft tissue disorders- Rheumatism (23216) | √ |  | **√** |  | X |
| Other soft tissue disorders (23140) | √ |  | **√** |  | X |
| Dorsopathies- Sciatica (23425) | √ |  | **√** |  | X |
| Dorsopathies­- Disc disorders (23171) | √ |  | **√** |  | X |
| Dorsopathies- Curvature of the spine (23424) | √ |  | **√** |  | X |
| Dorsopathies -Back pain/problems not elsewhere classified (23406) | √ |  | **√** |  | X |
| Osteoporosis (23455) | √ |  | **√** |  | X |
| Other diseases musculoskeletal system and connective tissue (23173) | √ |  | **√** |  | X |
| Symptoms, signs involving nervous and musculoskeletal system (23137) | √ |  | **√** |  | X |
| Urinary calculus (24721) | √ |  | X |  | X |
| Incontinence: urine (24696) | √ |  | X |  | X |
| Diseases of male genital organs (24814) | √ |  | X |  | X |
| Diseases of female pelvic organs and genital tract (24732) | √ |  | X |  | X |
| Other diseases of genito-urinary system (24203) | √ |  | X |  | X |
| Congenital malformations, deformations and chromosomal abnormalities -Of musculoskeletal system (25420) | √ |  | X |  | X |
| Other congenital malformations, deformations and chromosomal abnormalities (25054) | √ |  | X |  | X |
| Disability –not elsewhere classified (26017) | √ |  | X |  | X |
| Speech difficulties (26469) | √ |  | X |  | X |
| Fluid retention (non-circulatory) (26983) | √ |  | X |  | X |
| Allergy (undefined) (26056) | √ |  | X |  | X |
| Injuries –Fractures (26162) | √ |  | X |  | X |
| Injuries -Sprains, Strains and Tears of ligament, muscle or tendon (26167) | √ |  | X |  | X |
| Injuries -Injury joint, knee –not elsewhere classified (26172) | √ |  | X |  | X |
| Injury nerve (26502) | √ |  | X |  | X |
| Other injuries (26029) | √ |  | X |  | X |
| All other symptoms, signs and conditions (26001) | √ |  | X |  | X |
