# Supplementary material for: Cross-sectional examination of musculoskeletal conditions and multimorbidity: influence of different thresholds and definitions on prevalence and association estimates
Source: BMC Res Notes. 2017 Jan 18;10:51. doi: 10.1186/s13104-017-2376-4 (PMC5242059; doi:10.1186/s13104-017-2376-4)
Supplement: Supplementary file 3 — Additional file 3. Glossary of terms. [file 13104_2017_2376_MOESM3_ESM.docx]

**S3 Glossary of terms:**

**Nominal threshold**: the minimum number of conditions considered to constitute multimorbidity. We examine both a two condition and a three condition threshold.

**Operational definition:** the range of conditions included in any pre-specified list used to estimate the presence of multimorbidity.

**Condition abstraction:** the process of combining multiple related diagnoses into a single organ systems or domain when operationalising multimorbidity. For example some operational definitions of multimorbidity collate similar diagnoses into related condition categories based on organ systems affected and each affected organ system is counted once, while other definitions count each diagnosis individually regardless of whether they affect the same system.

**Operational definitions of multimorbidity:**

- **Survey-based:** an open-ended operational definition of multimorbidity, inclusive of all chronic conditions reported by survey respondents. The presence of each condition reported by respondents counted towards multimorbidity.
- **Policy-based:** an operational definition of multimorbidity restricted to chronic conditions identified as National Health Priority Areas for Australia, primarily abstracted into related categories. The presence of the following conditions/categories each count once towards multimorbidity: musculoskeletal conditions, diabetes, cancer, cardiovascular disease, asthma, chronic obstructive pulmonary disease, and mental health disorders.
- **Research-based:** an operational definition of multimorbidity based on a recent literature review by Diederichs et al (2011). The presence of the following conditions/categories each count once towards multimorbidity: cancer, diabetes mellitus, depression, hypertension, myocardial infarction, chronic ischemic heart disease, heart arrhythmias, heart insufficiency, stroke, chronic obstructive pulmonary disease, and arthritis.
